# An overview of cutaneous squamous cell carcinoma imaging diagnosis methods

**DOI:** 10.3389/fmed.2024.1388835

**Published:** 2024-04-25

**Authors:** Sorina Danescu, Mircea Negrutiu, Monica Focsan, Adrian Baican

**Affiliations:** ^1^Department of Dermatology, "Iuliu Hatieganu" University of Medicine and Pharmacy, Cluj-Napoca, Romania; ^2^Nanobiophotonics and Laser Microspectroscopy Center, Interdisciplinary Research Institute on Bio-Nano-Sciences, Babes-Bolyai University, Cluj-Napoca, Romania; ^3^Biomolecular Physics Department, Faculty of Physics, Babes-Bolyai University, Cluj-Napoca, Romania

**Keywords:** cutaneous squamous cell carcinoma, Dermoscopy, skin ultrasound, confocal microscopy, line-field confocal optical coherence tomography

## Abstract

Cutaneous squamous cell carcinoma, a type of non-melanoma skin cancer, is a form of keratinocyte carcinoma that stands as one of the most prevalent cancers, exhibiting a rising frequency. This review provides an overview of the latest literature on imaging methods for diagnosing squamous cell carcinoma (SCC) and actinic keratosis (AK). It discusses the diagnostic criteria, advantages, and disadvantages of various techniques such as dermatoscopy, skin ultrasound (US), *in vivo* and *ex-vivo* reflectance confocal microscopy (RCM), and line-field confocal optical coherence tomography (LC-OCT). These methods offer benefits including non-invasiveness, rapidity, comprehensive lesion imaging, and enhanced sensitivity, but face challenges like high costs and the need for specialized expertise. Despite obstacles, the use of these innovative techniques is expected to increase with ongoing technological advancements, improving diagnosis and treatment planning for keratinocyte carcinomas. Standardizing LC-OCT imaging algorithms for AK, Bowen’s disease, and SCC remains an area for further research.

## 1 Introduction

Cutaneous squamous cell carcinoma (cSCC) is the second most frequent form of cutaneous cancer with a high global growing incidence due to age (1). The clinical aspect of SCC is multiform and it varies according to histological subtype and tumor location (2). Usually an erythematous squamous plaque appears on photo-exposed areas on face, neck and forearms. Recurrence and metastasis are most commonly associated with a tumor diameter of more than 2 cm, thickness of more than 2 mm, perineural extensive invasion and poor differentiation in histology examinations (3). Most cases of cSCCs respond well to treatments such as surgical removal, photodynamic therapy, laser therapy, cryosurgery, and radiation. However, a small fraction, about 5% of patients, may have cSCCs that cannot be removed surgically, known as locally advanced (lacSCC) or metastatic (mcSCC) disease. Current research is focused on targeting the PD-L1/PD-1 axis to address lacSCC and mcSCC. Recently, Cemiplimab and Pembrolizumab received FDA approval for treating locally advanced and metastatic cSCCs, while ongoing studies are assessing the effectiveness and safety of Nivolumab and Ipilimumab for these conditions (4).

cSCC accounts for 20% of keratinocyte carcinoma (KC), with ratios of basal cell carcinoma (BCC) to cSCC ranging from 2 to 4:1. The European data on metastatic risk reveal a 2.1% cumulative incidence after a median follow-up of 15.2 months, with higher risk in males, older individuals, and certain anatomical locations (5).

Although comprehensive documentation of mortality rates is lacking, research findings indicate 5-year relative survival rates for localized cSCC, with percentages of 88% for women and 82% for men, and for advanced cSCC, with percentages of 64% for women and 51% for men (6).

Currently, biopsy and histopathologic examination serve as the gold standard for diagnosing skin cancer, but this approach has its drawbacks. Actinic keratoses (AK) often occur in multiple sites, making multiple biopsies impractical due to time, cost, and aesthetic concerns. Noninvasive imaging can guide biopsies effectively. Many dermatologists utilize dermoscopy as a rapid and low-cost way to assess lesions that look suspicious to the unaided eye. However, relying solely on clinical and dermoscopic data can be challenging, particularly for distinguishing AK from SCC. Large studies assessing diagnostic accuracy, especially in this context, are scarce. Dermoscopy also misses subclinical AK (7).

Recent advancements like reflectance confocal microscopy (RCM), optical coherence tomography (OCT), and high-frequency ultrasound (US) enhance skin cancer diagnosis accuracy.

The purpose of this review is to provide an update on the literature regarding the main methods of imaging diagnosis for squamous cell carcinoma and actinic keratosis, to highlight the diagnostic criteria, and to present the advantages and disadvantages of these methods. An electronic search of the literature was conducted using PUBMED and SCOPUS databases, encompassing articles published between inception and February 2024. The following search terms were used: “cutaneous squamous cell carcinoma” and cutaneous squamous carcinoma and imaging diagnosis.” Following the electronic search, bibliographies of included articles were reviewed and original articles and review articles were included.

### 1.1 Dermoscopy

Dermoscopy is a rapid and cost-effective method widely used by dermatologists to evaluate suspicious lesions and there is a growing evidence suggesting that dermoscopy improves early detection of skin cancer compared to naked-eye examination (7). Integration of dermoscopy into the diagnostic process also reduces the necessity for biopsies. However, dermoscopy is a subjective procedure requiring extensive training, posing a risk of misidentification of lesions.

Specific dermoscopic criteria for SCC, including keratin, scale, blood spots, white circles, white structureless zones, and perivascular white halos, have been established. For SCC, keratin and white circles exhibit diagnostic sensitivity and specificity rates of 79 and 87%, respectively (8).

Lallas et al. identified significant differences in the dermoscopic pattern of poorly differentiated SCC compared to well- and moderately differentiated tumors. White-colored criteria, including keratin, white circles, white halos, and structureless whitish areas, were associated with well- or moderately differentiated variants. Conversely, poorly differentiated SCC exhibited a predominantly red color due to dense vascularity, without keratin or other white-colored criteria.

The quantity and caliber of vessels were correlated with the differentiation grade. Tumors with vessels covering more than 50% of the lesion surface had a 30- to 120-fold increased likelihood of poor differentiation. The size of vessels emerged as a notable predictor of differentiation grade, with smaller sizes linked to a threefold higher probability of poor differentiation, whereas tumors with larger vessel sizes had an 83% lower likelihood of being poorly differentiated. Similar to a previous study, a central distribution of keratin proved to be a strong predictor of well-differentiated tumors (9).

In 2012 Zalaudek et al. proposed a progression model of AK into SCC *in situ* and invasive (10). The grading system for AK categorizes lesions based on their clinical and dermoscopic characteristics. Grade 1 lesions are clinically discernible but slightly palpable, displaying a red pseudonetwork pattern and discrete white scales under dermoscopy. Grade 2 lesions are thicker and easily visible and felt, featuring an erythematous background with white to yellow, keratotic follicular openings resembling a ‘strawberry pattern’. Grade 3 lesions are extremely thick and hyperkeratotic, clinically evident, and may exhibit enlarged follicular openings filled with keratotic plugs or marked hyperkeratosis presenting as white to yellow, structureless areas. Dermoscopy shows high diagnostic sensitivity and specificity for classical AK (98 and 95%, respectively) (11). Pigmented AK often displays a superficial brown network surrounding keratotic follicles, occasionally with red pseudonetwork and scales, while lichenoid AK may resemble lentigo maligna with grey dots, grey-brown lines, and asymmetrical pigmented follicular openings (12).

Non-Pigmented Intraepidermal Carcinoma (Bowens’ Disease, BD) is characterized by opaque, yellow to white scales, in addition to dotted and glomerular capillaries grouped in lines or clusters near the periphery (13). The diagnostic has a 98% sensitivity (14).

In Pigmented BD dermoscopic examination may reveal various structures, including thick, pigmented lines, brown spots, and/or structureless brown to grey regions (15). Alongside pigmented areas, pink, skin-colored, or white structureless regions, as well as coiled or dotted vessels, are commonly observed. An important diagnostic indicator for pigmented BD is the presence of radially oriented lines or dots arranged linearly at the periphery. Yang et al. identified two new dermoscopic signs: the double-edge sign, characterized by parallel pigmented edges at the lesion’s periphery (seen in 30.1% of lesions) and clusters of brown structureless areas, typically found around the lesion’s periphery (observed in 38.4% of lesions) (16).

In invasive SCC, vascular patterns are observed (linear-irregular and/or hairpin vessels) associated with indicators of keratinization (amorphous, structureless white to yellow areas or follicular openings with a targetoid appearance, characterized by an opaque, yellow center encircled by a white halo, named white circles) (10).

The various histological subtypes of SCC exhibit varying types and frequencies of vascular patterns and keratin clues.

Well-Differentiated SCC (Keratoacanthoma-type) is characterized by a central mass of keratin, appearing as an amorphous, structureless, white to yellow keratotic area, surrounded by elongated telangiectasias, described as linear, dull, red vessels with a large caliber and few branches. A polymorphous pattern including hairpin and dotted vessels may be also seen.

In a prospective study conducted by Phyne et al., 100 cases of keratoacanthoma (KA) and 410 cases of invasive SCC were analyzed. The study found that branching vessels were more common in KA compared to invasive SCC. However, the presence of pink within the tumor and the distribution of central versus peripheral tumor vessels were not identified as useful diagnostic features for distinguishing KA from SCC using dermoscopy (17).

Moderately differentiated SSC typically presents with peripheral hairpin vessels and diffuse, structureless, yellow to light brown, or white amorphous areas (keratin) that are often accompanied by extensive, variable areas of ulceration. Additional significant criteria include targetoid-appearing hair follicles (white circles), white, diffuse, structureless areas, masses of keratin interspersed with blood spots, and ulceration. The diagnostic sensitivity for keratin (structureless, white to yellow areas) has been reported to reach 78.7%, and the diagnostic specificity of the white circles is 86.9% (8).

Poorly differentiated subtypes frequently show no keratinization at all. Rather, they are identified by a variety of polymorphous vascular patterns, which include small-caliber linear vessels, hairpin vessels, or glomerular vessels against a reddish background. Occasionally, structureless white areas may be visible at the periphery, and these can be a crucial diagnostic indicator (12).

The occurrence of pigmented SCC (PSCC) varies significantly, with reported rates ranging from 0.01 to 7% of all SCCs in English literature. In his study, Corneli et al. highlight the specific features of PSCC, such as blue areas and linear polymorphous vessels around a hyperkeratotic region, also bluish diffuse pigmentation with central ulceration. Additionally, PSCC may exhibit dermoscopic features resembling those of melanocytic lesions, such as radial brown streaks and globules (18).

Dermoscopy reaches 79% sensitivity and 87% specificity in the diagnosis of SCC (19).

### 1.2 US

Skin ultrasound (US) is proven to be of assistance in evaluating size, thickness, tumor vascularization, relation with neighboring vascular structures and invasion. This information is essential before surgery for choosing the type of incision and evaluate conservative treatment options. Based on US characteristics (size, thickness, hyperechoic spots, posterior amplification and Doppler pattern) Chen Zt et al. demonstrated the value of HFUS in differentiating high risk basal cell carcinoma (BCC) and SCC (20). Another US difference between BCC and SCC is the disposition of the neovascularization. In SCC, the vascular pattern is increased diffusely throughout the mass, unlike BCC where the vascularization is less prominent and often located in the lower part of the lesion (21).

A study conducted by Bergón-Sendín M et al. on a group of 40 patients with SCC following a treatment with Methotrexate found positive correlation between ultrasound and histological diameter thickness evaluation (22). US utility in differential diagnosis between SCC, actinic keratoses and Bowen disease was evaluated by Zhu AQ et al. in a retrospective study of 160 patients. US characteristics for actinic keratoses were regular surface and irregular base. For Bowen disease, the US characteristics were crumpled surface and layer involvement confined by epidermis. For *in situ* SCC the US characteristics described were concave surface stratum corneum detachment, irregular based border and convex surface (23).

Keratoacanthoma are tumors that can be distinctly described through clinical examination and histopathology. Differentiating to SCC remains a controversial subject (24). The most common variant of keratoacanthoma is the solitary type with irregular shape, variable dimension and a characteristic evolution that can be divided in a proliferative, stationary and regressive phase (24–26). On US imaging keratoacanthoma is a hypoechoic lesion with relatively good definition, homogenous pattern, with hypoechoic stroma and a hyperechoic keratin crater (26).

### 1.3 Confocal microscopy

*In vivo* reflectance confocal microscopy (RCM) is a non-invasive diagnostic technique that makes several high-resolution two-dimensional images at various skin depths (from the stratum corneum to the papillary dermis). In order to increase diagnostic precision for both melanocytic and non-melanocytic cutaneous lesions as well as certain non-neoplastic disorders, RCM is being used more and more in clinical practice (27).

RCM has proven its usefulness in differentiating between invasive and *in situ* SCC. Manfredini M et al. propose the following criteria for invasive SCC: presence of erosion/ulceration, architectural disarrangement, speckled nucleated cells in the dermis, and absence of hyperkeratosis (28). The most described RCM aspects found in SCC are: scale, hyperkeratosis, parakeratosis, architectural disarray in stratum granulosum, atypical honeycomb pattern in stratum granulosum, atypical honeycomb pattern or architectural disarray in stratum spinosum, round nucleated cells, dilated blood vessels, increased number of blood vessels, nest-like structures in superficial dermis, pleomorphic nucleated cells in superficial dermis (29).

The RCM of *in-situ* SCC resembles an unusual honeycomb pattern, with non-confluent spindle-shaped cells and delicate dendritic branches penetrating the suprabasal epidermis, which is consistent with Langerhans cells. Furthermore, numerous bright dermo-epidermal junction (DEJ) -edged papillae are present, primarily along the margin of the lesion. These papillae are tiny, spherical, and have enlarged interpapillary gaps (30).

According to Peppelman et al. the diameter of blood vessels and the number of blood vessels visualized with RCM is increased in the case of AK and SCC compared to healthy skin, being higher in the case of SCC (31).

RCM has proven useful in differentiating actinic cheilitis (AC) from SCC. Lupu M et al. propose the following characteristics for AC: atypical honeycomb pattern and the presence of target cells in the epidermis (32). By preventing needless biopsies, particularly in lesions with persistent residual postinflammatory erythema, RCM may be a useful tool in the diagnosis of *in situ* SCC and in tracking the effectiveness of nonsurgical treatment (33).

*Ex-vivo* confocal microscopy was created to help Mohs surgery by providing a quick perioperative evaluation of the surgical margins in addition to enabling prompt identification of recently removed tissues. As *ex-vivo* confocal microscopy determines residual tumour tissue more quickly than histopathologic evaluation of frozen sections, it may be a useful and expedient substitute for traditional Mohs surgery. Additionally, it prevents material waste that could result in sections that run the risk of significant degradation for the future examination using conventional pathology and enables digital sectioning of the specimens without causing injury to the tissue (34).

A relatively new technique is *ex vivo* fluorescence confocal microscopy (FCM). This allows the evaluation of the tumor and the resection margins directly in freshly excised tissue, with a resolution comparable to histology, but in a shorter time. Characteristics such as fluorescence, tumor silhouette, keratin pearls (ie, concentric laminated low fluorescent whorls of keratinized squames), nuclear pleomorphism (ie, variation in size, shape, and fluorescence of the keratinocytes), and keratin formation can categorize SCC as being well, moderately, or poorly differentiated (35).

### 1.4 Line-field confocal optical coherence tomography

Line-field confocal optical coherence tomography (LC-OCT) is a novel noninvasive technique for skin imaging that combines the advantages of optical coherence tomography (OCT) and reflectance confocal microscopy (RCM) in terms of spatial resolution, penetration depth, and image orientation. LC-OCT achieves superior resolution (~1 μm) compared to OCT and greater penetration depth (~500 μm) than RCM. Additionally, LC-OCT enables the simultaneous generation of vertical and horizontal images in real time (36).

Using LC-OCT, Ruini et al. visualized AKs and their main histopathological features. The sample size of AK subtypes was limited, but they observed the flattened rete in hypertrophic AKs, thinned epidermis in atrophic AKs, and full-thickness keratinocyte dysplasia with rounded contours in bowenoid AKs. In early AKs, dermoscopic structureless red areas interrupted by follicular openings corresponded to dilated and tortuous hyporeflective vessels in the superficial dermis and hyperreflective follicular hyperkeratosis.

Within BD, the thickened epidermis is characterized by enlarged, atypical keratinocytes forming round contours throughout all layers, termed the bowenoid pattern.

In both BD and AK, the dermoepidermal junction (DEJ) appeared well-preserved, presenting as a distinct darker band separating the basal keratinocytes from the bright dermal collagen.

SCC presented disorganized clusters of large polygonal cells with irregular shapes, accompanied by round, bright, homogeneous structures indicative of horn pearls. When tumor strands and masses were apparent, the dermis showed signs of elastosis and collagen changes along with increased and irregular vascularization (including dilated arteries and neoangiogenesis). LC-OCT aids in accurate classification of keratinocyte carcinoma subtypes, achieving a 100% correct classification rate (37).

While LC-OCT offers benefits in direct navigation and cellular resolution imaging, it may miss deeper layers in hyperkeratotic lesions.

Ruini et al. demonstrated that based on the histological PRO classification of AK, LC-OCT can reliably assess the basal keratinocyte development pattern *in vivo* (38). PRO classification delineates different stages of downward extension of basal keratinocytes into the papillary dermis. Specifically, PRO I involves the “crowding” of atypical keratinocytes in the basal layer, PRO II entails their “budding” in round nests into the upper papillary dermis, and PRO III is characterized by “papillary sprouting,” where spikes of atypical keratinocytes protrude into the dermis, thicker than the overlying epidermis (39).

Donelli et al. showed in their study that LC-OCT exhibited higher specificity and a slightly increased sensitivity compared to dermoscopy in diagnosis of skin carcinomas. LC-OCT showed superior capability in ruling out malignancy rather than providing a precise diagnosis (40).

In a study conducted by Cinotti et al., evaluations of AK and SCC by RCM and LC-OCT were compared. Both techniques showed a significant level of agreement, the majority of the tumors had an irregular epidermis, but LC-OCT provided a clearer picture of parakeratosis, dyskeratotic keratinocytes, and both linear and glomerular vasculature than RCM did (*p* < 0.001). In more than half of the cases, erosion or ulceration was seen using both techniques, and there was a significant level of agreement (41).

## 2 Discussion

By employing specific dermatoscopic patterns, it becomes feasible to differentiate SCC from other nonmelanocytic tumors. Moreover, the invasive progression of precursor lesions can be identified earlier and with increased certainty within the framework of a “progression model.” Skin US has demonstrated its utility in assessing dimensions, thickness, tumor vascularity, proximity to adjacent vascular structures, and invasion. *In vivo* RCM is a non-invasive diagnostic method that captures high-resolution skin images at various depths, effectively distinguishing between invasive and *in situ* SCC. *Ex-vivo* RCM can rapidly identify residual tumor tissue, offering an efficient alternative to traditional Mohs surgery. LC-OCT enables simultaneous real-time imaging in both vertical and horizontal orientations, aiding in precise keratinocyte carcinoma subtype classification and offering greater penetration depth than RCM.

**Figure 1:**
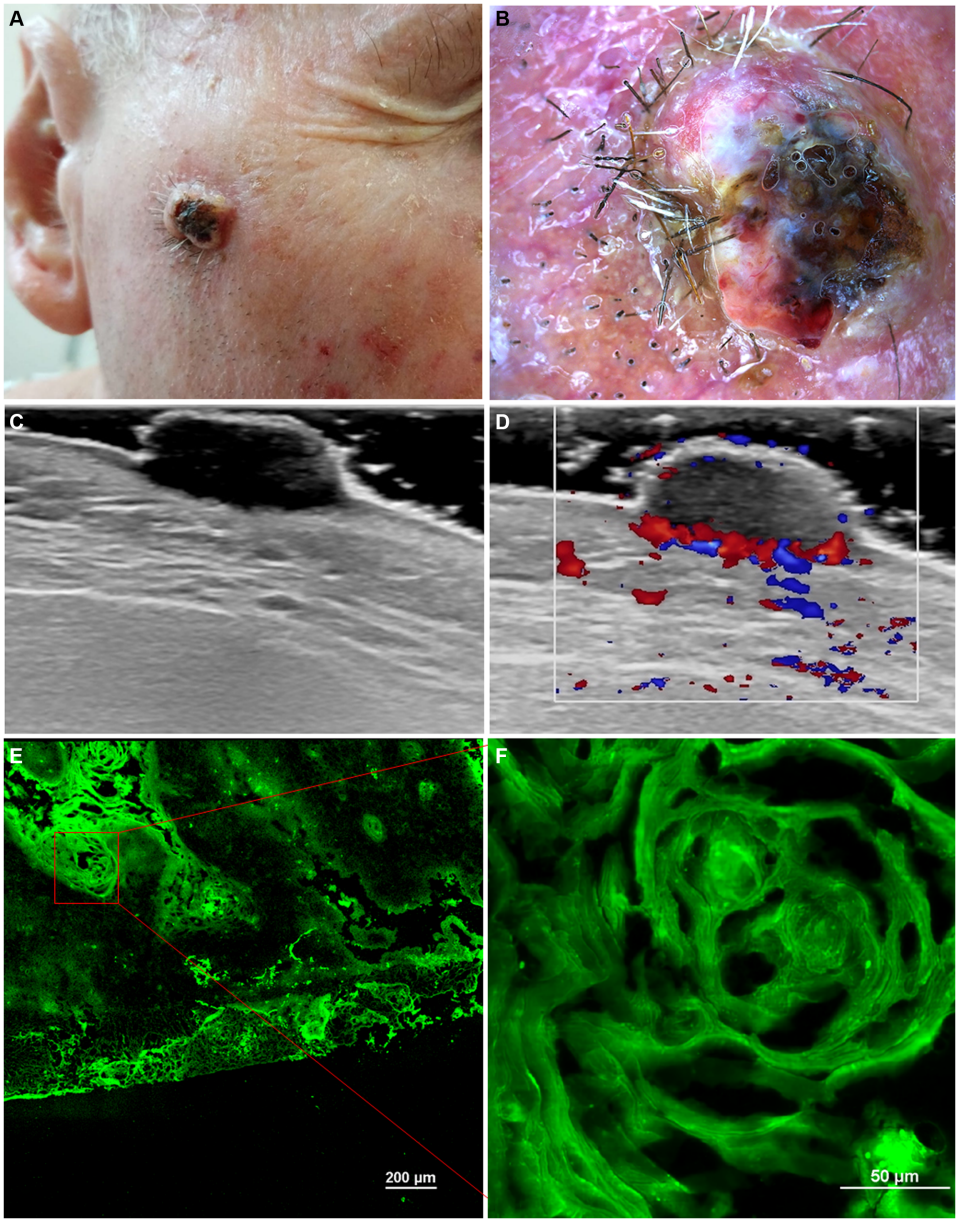
Clinical picture of well-differentiated keratinizing squamous cell carcinoma **(A)**. Dermoscopy shows white structureless areas, surface keratin, ulceration and looped vessels **(B)**. Ultrasonography (US) shows a hypoechoic lesion, imprecisely delimited, located at the level of the epidermis and dermis **(C)**. Doppler mode shows an increase in tumor and peripheral vascularity **(D)**. *Ex vivo* confocal microscopy with fluorescence (FCM) shows a highly fluorescent area with keratin pearls **(E, F)**.

A summary of the main imagistic aspects identified in actinic keratosis, Bowen’s Disease and invasive Squamous Cell Carcinoma are presented in Table 1 and some of the imagistic features of a well differentiated squamous cell carcinoma are illustrated in Figure 1. The patient’s informed consent was obtained.

**Table 1:** Summary of the main imagistic aspects in actinic keratosis, Bowen’s disease and invasive squamous cell carcinoma, advantages and disadvantages of each technique.

	**Actinic keratosis**	**Bowen’s disease**	**Invasive squamous cell carcinoma**	**Advantages**	**Disadvantages**
**Dermatoscopy features**	Erythema-reticular vessels surrounding follicular openings, strawberry pattern, rosettes ^11^	opaque, yellow to white scales, dotted and glomerular capillaries grouped in lines or clusters near the periphery ^14^	amorphous, structureless white to yellow areas or follicular openings with a targetoid appearance, linear-irregular and/or hairpin vessels ^11^	rapid and cost-effective; widely used by dermatologists; sensitivity and specificity rates of 79 and 87% for SCC ^20^	subjective procedure; requiring extensive training
**Ultrasound features**	regular surface and irregular base ^24^	crumpled surface and layer involvement confined by epidermis.^24^	concave surface, stratum corneum detachment, Irregular based border, the vascular pattern is increased diffusely throughout the mass^24^	essential before surgery for choosing the type of incision; evaluating the size and depth of tumors ^21^	depends on the operator; subjective technique, time-consuming, requires a lot of experience, difficult in assessing concave areas, internal angle of the orbit, requires high-frequency probes especially for lesions under 1 mm.
**Reflectance confocal microscopy features**	atypical honeycomb pattern and the presence of target cells in the epidermis ^33^	honeycomb pattern, with non-confluent spindle-shaped cells and delicate dendritic branches penetrating the suprabasal epidermis, numerous bright dermo-epidermal junction edged papillae ^31^	hyperkeratosis, parakeratosis, atypical honeycomb pattern in stratum granulosum, atypical honeycomb pattern in stratum spinosum, round nucleated cells, dilated and increased number of blood vessels, nest-like structures in superficial dermis ^30^	differentiating between invasive and *in situ* SCC; offers horizontal sections; evaluation of therapeutic effects ^34^	time consuming, expensive method, offers an imaging depth less than 250 mm; requires a lot of experience, difficult in assessing concave areas
**Line-field confocal optical coherence tomography features**	flattened rete in hypertrophic AKs, thinned epidermis in atrophic AKs, full-thickness keratinocyte dysplasia with rounded contours in bowenoid AKs ^38^	enlarged, atypical keratinocytes forming round contours throughout all layers ^38^	disorganized clusters of large polygonal cells with irregular shapes, round, bright, homogeneous structures indicative of horn pearls, increased and irregular vascularization ^38^	greater penetration depth (~500 μm) than RCM; aids in accurate classification of keratinocyte carcinoma subtypes, generation of vertical and horizontal images in real time ^37^	may miss deeper layers in hyperkeratotic lesions, expensive technique, novel technique, that requires experience.

These novel approaches offer advantages including rapidity, non-invasiveness, comprehensive lesion imaging, remote diagnostic capabilities, and enhanced sensitivity. Nevertheless, their widespread adoption is hindered by factors such as high expenses, requisite expertise of operators and interpreters, anatomical constraints, and limited specificity. The utilization of these innovative techniques for diagnosing skin cancer is anticipated to rise as ongoing research refines their technology and diagnostic precision. The incorporation of clinical, dermoscopic, and imaging data improves the diagnosis and treatment planning for keratinocyte carcinomas. Further research is necessary to standardize line-field confocal optical coherence tomography (LC-OCT) imaging algorithms for actinic keratoses, Bowen’s disease, and squamous cell carcinomas.

There are few review articles in the literature providing an overview of imaging diagnostic techniques in squamous cell carcinoma. In recent years, numerous imaging diagnostic techniques have emerged, increasing the specificity and sensitivity of diagnosis. In many cases, clinical and dermoscopic diagnosis are sufficient for a probable diagnosis, but additional techniques are needed to improve diagnostic accuracy. Moreover, to assess the size and depth of the lesion, supplementary techniques are required for surgical intervention planning. In the future, more diagnostic techniques with increased sensitivity and specificity, easy to use and cost-accessible, will certainly emerge, and an artificial intelligence program will be developed to integrate and analyze data from these techniques. The main strengths of the manuscript are: it provides the main features of the each diagnostic technique, based on the most important references published in the literature, the advantages and disadvantages of each technique. The limitations of the manuscript: there is a limited depth on standardization. While the manuscript acknowledges the importance of standardizing LC-OCT imaging algorithms for AK, Bowen’s disease, and SCC, it could provide more detailed insights into the specific challenges and potential strategies for achieving standardization. Another limitation could be the potential bias: depending on the selection and interpretation of the literature reviewed, there may be a risk of bias towards certain imaging techniques or approaches.

After an exhaustive study of the literature, we found relatively few studies on cutaneous ultrasound in squamous cell carcinoma. In this review, we included all the research we found and did not notice the presentation of the US diagnostic criteria for CSC, as well as the sensitivity and specificity of this imaging technique. If criteria were found for BCC (relatively well-defined tumors, with variable vascularization and with presence of hyperechoic spots) additional studies are needed among CSC. US patterns could be identified depending on the histological type and specific anatomical location of CSC. Also, we did not find studies that show the correlation between the histological thickness and US thickness, considering that the treatment is guided according to the tumor thickness. Being an invasive tumor both locally and at a distance, future perspectives could show the role of US in the loco-regional evaluation of the disease. In the case of superficial carcinomas, the response to topical therapy could be evaluated by measuring the tumor thickness before and after treatment.

In conclusion, among the diagnostic methods discussed in this article, we believe that significant progress has been made by confocal microscopy, both in diagnosing skin cancers and non-tumor pathology. However, the greatest potential to revolutionize dermatology and skin cancer lies with LC-OCT, both in terms of precise diagnosis and evaluating tumor margins for Mohs surgery. Nevertheless, there is a need for improvement in skin penetrability, cost accessibility, and easier-to-handle equipment.

## Author contributions

SD: Writing – review & editing, Conceptualization. MN: Writing – review & editing. MF: Writing, Formal analysis. AB: Writing – review & editing.
